# Analysis of related factors of CRA in lung cancer patients with different serum iron levels: A retrospective cohort study

**DOI:** 10.1002/cam4.7147

**Published:** 2024-04-01

**Authors:** Quan‐yao Li, Wen‐xiao Yang, Hui Liu, Jia‐lin Yao, Qin Wang, Dan Lin, Jun Shi

**Affiliations:** ^1^ Yueyang Hospital of Integrated Traditional Chinese and Western Medicine Affiliated to Shanghai University of Traditional Chinese Medicine Shanghai China; ^2^ Department of Traditional Chinese Medicine Shanghai Fourth People's Hospital Affiliated to Tongji University of Medicine Shanghai China

**Keywords:** cancer‐related anemia, influencing factors, lung cancer, nomogram model, serum iron

## Abstract

**Background:**

Serum iron, an essential component of hemoglobin (Hb) synthesis in vivo, is a crucial parameter for evaluating the body's iron storage and metabolism capacity. Iron deficiency leads to reduced Hb synthesis in red blood cells and smaller red blood cell volume, ultimately resulting in iron‐deficiency anemia. Although serum iron cannot independently evaluate iron storage or metabolism ability, it can reflect iron concentration in vivo and serve as a good predictor of iron‐deficiency anemia. Therefore, exploring the influence of different serum iron levels on anemia and diagnosing and treating iron deficiency in the early stages is of great significance for patients with lung cancer.

**Aim:**

This study aims to explore the related factors of cancer‐related anemia (CRA) in lung cancer and construct a nomogram prediction model to evaluate the risk of CRA in patients with different serum iron levels.

**Methods:**

A single‐center retrospective cohort study was conducted, including 1610 patients with lung cancer, of whom 1040 had CRA. The relationship between CRA and its influencing factors was analyzed using multiple linear regression models. Lung cancer patients were divided into two groups according to their serum iron levels: decreased serum iron and normal serum iron. Each group was randomly divided into a training cohort and a validation cohort at a ratio of 7:3. The influencing factors were screened by univariate and multivariate logistic regression analyses, and nomogram models were constructed. The area under the receiver operating characteristic (ROC) curve, calibration curve, and decision curve analysis (DCA) were used to evaluate the models.

**Results:**

CRA in lung cancer is mainly related to surgery, chemotherapy, Karnofsky Performance Status (KPS) score, serum iron, C‐reactive protein (CRP), albumin, and total cholesterol (*p* < 0.05). CRA in lung cancer patients with decreased serum iron is primarily associated with albumin, age, and cancer staging, while CRA in lung cancer patients with normal serum iron is mainly related to CRP, albumin, total cholesterol, and cancer staging. The area under the ROC curve of the training cohort and validation cohort for the prediction model of lung cancer patients with decreased serum iron was 0.758 and 0.760, respectively. Similarly, the area under the ROC curve of the training cohort and validation cohort for the prediction model of lung cancer patients with normal serum iron was 0.715 and 0.730, respectively. The calibration curves of both prediction models were around the ideal 45° line, suggesting good discrimination and calibration. DCA showed that the nomograms had good clinical utility.

**Conclusion:**

Both models have good reliability and validity and have significant clinical value. They can help doctors better assess the risk of developing CRA in lung cancer patients. CRP is a risk factor for CRA in lung cancer patients with normal serum iron but not in patients with decreased serum iron. Therefore, whether CRP and the inflammatory state represented by CRP will further aggravate the decrease in serum iron levels, thus contributing to anemia, warrants further study.

## 1 INTRODUCTION

Lung cancer is one of the most common malignant tumors, with an incidence second only to breast cancer and the highest mortality rate among all solid tumors worldwide.^1^ The five‐year survival rate remains below 20%.^2^, ^3^ CRA is a common complication of tumors that is often overlooked. Its main manifestations are decreased red blood cells, hemoglobin (Hb) concentration, or red blood cell volume in peripheral blood. CRA is also common in lung cancer patients, with epidemiological investigations revealing an incidence as high as 32.09%.^4^ The occurrence and development of CRA seriously affect the prognosis of lung cancer patients. Studies have shown that CRA has become an independent risk factor endangering the survival rate and quality of life of lung cancer patients.^5^ Tumors can induce anemia and anemia can lead to hypoxia in tumor cells, induce changes in tumor gene expression, and inhibit tumor cell apoptosis, thereby enhancing their invasion ability and leading to the failure of antitumor treatment.^6^, ^7^ Additionally, CRA can cause various related symptoms,^8^ which seriously affect the efficacy of antitumor treatment and patients' quality of life. Current studies show that CRA in lung cancer is closely related to cancer staging, type, disease course, and therapeutic regimen,^9^, ^10^ but a risk factor prediction model is lacking.

Iron deficiency, which has a high incidence, is a common complication of tumors and an accompanying symptom of CRA. Iron deficiency is a continuous spectrum ranging from iron storage deficiency to iron‐deficient erythropoiesis, which eventually leads to iron‐deficiency anemia.^11^ Serum iron is an important component of Hb synthesis in the body, representing the iron concentration and occupying an indispensable position in iron deficiency. In the past, iron deficiency was thought to be related to malnutrition and was given more attention in digestive tract tumors. However, few studies have been conducted on CRA in lung cancer, with most studies limited to exploring its influencing factors and no effective prediction model established. Therefore, the significance of iron content represented by serum iron in the formation of CRA in lung cancer remains unclear. Based on univariate and multivariate logistic regression analyses of CRA‐related factors in lung cancer, this study established nomogram models to predict the CRA of decreased and normal serum iron levels in lung cancer to provide a scientific basis for clinical treatment.

## 2 METHODS

### 2.1 Research object

This study is a retrospective cohort study. From September 2017 to August 2022, patients with lung cancer were collected from the oncology department of Yueyang Hospital of Integrated Traditional Chinese and Western Medicine affiliated with Shanghai University of Traditional Chinese Medicine.

#### 2.1.1 Diagnostic criteria

The anemia standard refers to the World Health Organization's anemia classification standard in the Clinical Practice Guide of Tumor‐related Anemia (2022 edition) published by the China Society of Clinical Oncology (CSCO)^12^: Grade 0 (Normal value): adult males should not have an Hb level of less than 130 g/L, nonpregnant adult females should not have an Hb level of less than 120 g/L, and pregnant adult females should not have an Hb level of less than 110 g/L; Grade 1 (Mild anemia): 110 g/L‐Normal value; Grade 2 (Moderate anemia): 80 g/L‐109 g/L; Grade 3 (Severe anemia): < 80 g/L.

According to the Standard of Diagnosis and Curative Effect of Hematological Diseases, serum iron levels <8.95 μmol/L indicate decreased serum iron levels.^13^

#### 2.1.2 Inclusion criteria

A patient who meets the following criteria should be included:

1. Diagnosed with lung cancer by pathological cytology or histology. Patients with lung cancer CRA need to meet the above anemia standards; Patients with decreased serum iron in lung cancer need to meet the above decreased serum iron standard criteria.
2. The patient is between the ages of 18 and 90 years old and gender is not limited.
3. Patients who have complete clinical data.

#### 2.1.3 Exclusion criteria

1. Multiple primary tumor.
2. Prior history of tumors.
3. Anemia caused by nontumor related reasons such as aplastic anemia, renal anemia, and autoimmune diseases.

### 2.2 Data collection

Relevant case data of the patients at their hospital admission are collected, including: (1) Basic patient information: gender and age; (2) Clinical data: cancer staging, pathological type, surgery, radiotherapy, chemotherapy, targeted therapy, immunotherapy, KPS score; (3) Laboratory indexes: Hb, serum iron, CRP, albumin, and total cholesterol.

### 2.3 Research methods

(1) A multiple linear regression model is used to analyze the relationship between CRA in lung cancer and its influencing factors.

(2) Cohort definitions and variable records: Patients with decreased or normal serum iron levels in lung cancer are randomly divided into a training cohort and a validation cohort at a 7:3 ratio using SPSS 26.0. The training cohort is used to construct the model, while the validation cohort is used to validate the results obtained using the training cohort. The influencing factors are screened by univariate and multivariate logistic regression analyses, and nomogram models are constructed respectively. Based on different nomogram models, the area under the ROC curve is drawn to evaluate the discrimination of the models, the calibration curve is drawn to evaluate the calibration degree of the models, and the DCA is drawn to evaluate the clinical practicability.

### 2.4 Statistical analysis

SPSS 26.0 and R 4.4.2 software are used for statistical analysis of the data. Measurement data are expressed as mean ± standard deviation, and t‐tests are used to compare differences between two groups. Chi‐square tests are used for counting data. Multivariate logistic regression analysis is used to screen related factors. R 4.2.2 software is used to build nomogram models. ROC curves and calibration curves are drawn by the rms package, and DCA curves are drawn by the rmda package.

## 3 RESULTS

**FIGURE 1:**
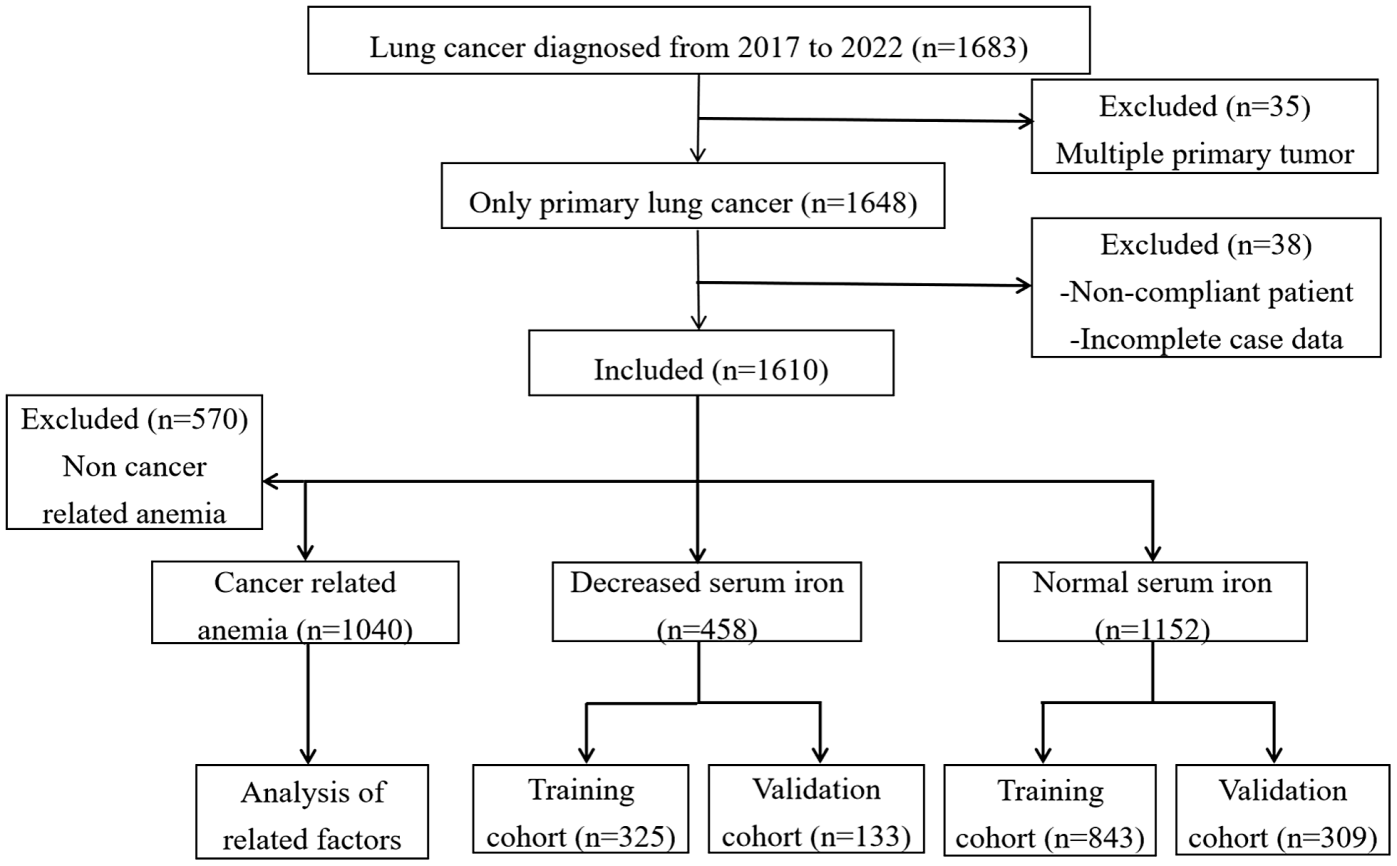
Flow diagram.

A total of 1610 patients with lung cancer were included. According to the inclusion and exclusion criteria, 1040 patients with lung cancer CRA were included (64.6%), including 390 patients with decreased serum iron (37.5%) and 650 patients with normal serum iron (62.5%). Among the 1610 patients with lung cancer, there were 458 patients with decreased serum iron (28.45%) and 1152 patients with normal serum iron (71.55%). See Figure 1.

### 3.1 CRA analysis of lung cancer

#### 3.1.1 Analysis of related factors of lung cancer CRA

Statistical analysis shows that there is no statistical difference in the effects of gender, age, radiotherapy, and immunotherapy on different degrees of CRA; the effects of different cancer staging, pathological types, surgery, chemotherapy, and targeted therapy on different degrees of CRA are statistically different. Taking the KPS score equal to 70 as the boundary, the influence of KPS on different degrees of anemia is statistically different. See Table 1.

**TABLE 1:** Analysis of Related Factors of CRA in Lung Cancer.

Items	Characteristic	Case	Grade 1	Grade 2	Grade 3	*χ* ^2^	*p*
Gender	Male	624	370	219	35	1.874	0.392
	Female	416	257	143	16		
Age	≤60	242	160	71	11	4.569	0.102
	>60	798	467	291	40		
Cancer staging**	I	224	176	47	1	59.168	<0.01
	II	166	106	51	9		
	III	191	113	73	5		
	IV	459	232	191	36		
Pathological type**	Adenocarcinoma	752	478	247	27	18.558	<0.01
	Squamous carcinoma	209	105	87	17		
	Small cell carcinoma	56	32	19	5		
	Others (large cell carcinoma、sarcomatoid carcinoma)	23	12	9	2		
Surgery**	Yes	563	334	215	14	18.844	<0.01
	No	477	293	147	37		
Radiotherapy	Yes	142	75	57	10	4.360	0.113
	No	898	551	305	41		
Chemotherapy**	Yes	534	290	210	34	454.217	<0.01
	No	506	337	152	17		
Targeted therapya*	Yes	192	100	83	9	7.450	<0.05
	No	848	527	279	42		
Immunotherapy	Yes	43	26	14	3	0.458	0.795
	No	997	601	348	48		
KPS**	<70	40	11	17	12	61.554	<0.01
	≥70	1000	616	345	39		

* *p* < 0.05.

** *p* < 0.01.

#### 3.1.2 Anemia of CRA patients with lung cancer and related laboratory examination indexes

Statistical analysis shows that there are significant differences in Hb, serum iron, C‐reactive protein, albumin, and total cholesterol in 1040 patients with lung cancer CRA (*p* < 0.01). See Table 2.

**TABLE 2:** Anemia of CRA Patients with Lung Cancer and Related Laboratory Examination Indicators.

Laboratory examination indicators	Grade 1	Grade 2	Grade 3	*F*	*p*
HB**	118.81 ± 5.55	98.80 ± 8.00	66.51 ± 10.27	768.105	<0.01
Serum iron**	13.07 ± 6.16	9.57 ± 7.24	6.76 ± 5.93	156.527	<0.01
CRP**	17.52 ± 33.97	35.99 ± 45.09	73.30 ± 52.63	126.526	<0.01
Albumin**	37.72 ± 4.38	34.71 ± 4.84	30.66 ± 5.21	142.038	<0.01
Total cholesterol**	4.67 ± 1.84	4.21 ± 0.96	3.63 ± 1.06	66.977	<0.01

** *p* < 0.01.

#### 3.1.3 Multivariate analysis of CRA related factors in lung cancer

Multiple linear regression is used to analyze the related factors of CRA in lung cancer. Hb is used as the dependent variable, and cancer staging, pathological type, surgery, chemotherapy, targeted therapy, KPS score, and related examination indicators are used as independent variables. Hypothesis test: *F* = 25.838, *p* < 0.001, which shows that the fitted regression equation has significant statistical significance. Collinearity test: VIF <5, indicating that the results are accurate and reliable. The results show that CRA in lung cancer is mainly related to serum iron, CRP, albumin, total cholesterol, KPS score, surgery, and chemotherapy (*p* < 0.05). See Table 3.

**TABLE 3:** Multiple linear regression analysis of CRA related factors in lung cancer.

Items	Characteristic	Regression coefficient	Standard error	Standard regression coefficient	*t*	*p*	95.0% CI	VIF
Serum iron**	Serum iron**	0.295	0.067	0.133	4.408	<0.01	0.164 ~ 0.426	1.221
CRP**	CRP**	−0.035	0.012	−0.097	−2.859	<0.01	−0.059 ~ −0.011	1.526
Albumin**	Albumin**	0.727	0.102	0.239	7.102	<0.01	0.526 ~ 0.928	1.512
Total cholesterol**	Total cholesterol**	0.763	0.267	0.08	2.862	<0.01	0.24 ~ 1.287	1.043
KPS*	KPS*	0.117	0.039	0.087	3.037	<0.01	0.042 ~ 0.193	1.09
Pathological type	Adenocarcinoma	0						
	Squamous carcinoma	0.191	1.111	0.005	0.172	0.863	−1.988 ~ 2.371	1.16
	Small cell carcinoma	0.917	1.929	0.014	0.476	0.634	−2.867 ~ 4.702	1.109
	Others (large cell carcinoma、sarcomatoid carcinoma)	3.629	2.856	0.035	1.271	0.204	−1.974 ~ 9.233	1.032
Cancer staging	Cancer staging	−0.337	0.447	−0.027	−0.753	0.451	−1.214 ~ 0.54	1.684
Surgery**	Yes	3.256	0.953	0.107	3.418	<0.01	1.387 ~ 5.125	1.319
	No	0						
Chemotherapy**	Yes	−2.771	0.929	−0.092	−2.982	<0.01	−4.594 ~ −0.948	1.262
	No	0						
Targeted therapy	Yes	−0.056	1.185	−0.001	−0.047	0.963	−2.382 ~ 2.27	1.238
	No							

* *p* < 0.05.

** *p* < 0.01.

### 3.2 Analysis of related factors of decreased serum iron and normal serum iron patients in lung cancer and construction of nomogram prediction model

#### 3.2.1 Clinical features

A total of 458 patients (28.45%) with decreased serum iron levels in lung cancer are randomly divided into 7:3, including 325 patients in the training cohort (70.96%) and 133 patients in the validation cohort (29.04%). Thousand one hundred and fifty‐two patients with normal serum iron levels in lung cancer (71.55%) are randomly divided into 7:3, including 843 patients in the training cohort (73.18%) and 309 patients in the validation cohort (26.82%). See Table 4.

**TABLE 4:** Clinical Characteristics of Lung Cancer Patients with Decreased Serum Iron.

Items	Characteristic	Patients with decreased serum iron in lung caner				Patients with normal serum iron in lung cancer			
		Training cohort (*n* = 325)		Validation cohort (*n* = 133)		Training cohort (*n* = 843)		Validation cohort (*n* = 309)	
		CRA [*n* (%)]	Non‐CRA [*n* (%)]	CRA [*n* (%)]	Non‐CRA [*n* (%)]	CRA [*n* (%)]	Non‐CRA [*n* (%)]	CRA [*n* (%)]	Non‐CRA [*n* (%)]
Gender	Male	204 (62.77)	23 (7.08)	68 (51.13)	11 (8.27)	257 (30.49)	163 (19.34)	95 (30.74)	54 (17.48)
	Female	74 (22.77)	24 (7.38)	44 (33.08)	10 (7.52)	214 (25.39)	209 (24.79)	84 (27.18)	76 (24.60)
Age	≤60	56 (17.23)	18 (5.54)	23 (17.29)	9 (6.77)	121 (14.35)	127 (15.07)	42 (13.59)	48 (15.53)
	>60	222 (68.31)	29 (8.92)	89 (66.92)	12 (9.02)	350 (41.52)	245 (29.06)	137 (44.34)	82 (26.54)
Cancer staging	I	41 (12.62)	25 (7.69)	17 (12.78)	11 (8.27)	118 (14.00)	215 (25.50)	48 (15.53)	77 (24.92)
	II	41 (12.62)	5 (1.54)	11 (8.27)	4 (3.01)	90 (10.68)	49 (5.81)	24 (7.77)	15 (4.85)
	III	57 (17.54)	4 (1.23)	17 (12.78)	2 (1.50)	89 (10.56)	38 (4.51)	28 (9.06)	11 (3.56)
	IV	139 (42.77)	13 (4.00)	67 (50.38)	4 (3.01)	174 (20.64)	70 (8.30)	79 (25.57)	27 (8.74)
Pathological type	Adenocarcinoma	177 (54.46)	46 (14.15)	73 (54.89)	20 (15.04)	369 (43.77)	330 (39.15)	33 (10.68)	117 (37.86)
	Squamous carcinoma	77 (23.69)	1 (0.31)	30 (22.56)	1 (0.75)	70 (8.30)	38 (4.51)	32 (10.36)	10 (3.24)
	Small cell carcinoma	16 (4.92)	0 (0)	6 (4.51)	0 (0)	23 (2.73)	2 (0.24)	11 (3.56)	1 (0.32)
	Others	8 (2.46)	0 (0)	3 (2.26)	0 (0)	9 (1.07)	2 (0.24)	3 (0.97)	2 (0.65)
Surgery	Yes	109 (33.54)	32 (9.85)	48 (36.09)	17 (12.78)	295 (34.99)	305 (36.18)	111 (35.92)	107 (34.63)
	No	169 (52.00)	15 (4.62)	64 (48.12)	4 (3.01)	176 (20.88)	67 (7.95)	68 (22.01)	23 (7.44)
Radiotherapy	Yes	42 (12.92)	5 (1.54)	13 (9.77)	1 (0.75)	63 (7.47)	31 (3.68)	24 (7.77)	10 (3.24)
	No	236 (72.62)	42 (12.92)	99 (74.44)	20 (15.04)	408 (48.40)	341 (40.45)	155 (50.16)	120 (38.83)
Chemotherapy	Yes	95 (29.23)	8 (2.46)	31 (23.31)	7 (5.26)	117 (13.88)	123 (14.59)	54 (17.48)	39 (12.62)
	No	183 (56.31)	39 (12.00)	81 (60.90)	14 (10.53)	354 (41.99)	249 (29.54)	125 (40.45)	91 (29.45)
Targeted therapy	Yes	53 (16.31)	8 (2.46)	24 (18.05)	5 (3.76)	81 (9.61)	51 (6.05)	34 (11.00)	19 (6.15)
	No	225 (69.23)	39 (12.00)	88 (66.17)	16 (12.03)	390 (46.26)	321 (38.08)	145 (46.93)	111 (35.92)
Immunotherapy	Yes	14 (4.31)	5 (1.54)	2 (1.50)	1 (0.75)	20 (2.37)	8 (0.95)	7 (2.27)	2 (0.65)
	No	264 (81.23)	42 (12.92)	110 (82.71)	20 (15.04)	451 (53.50)	364 (43.18)	172 (55.66)	128 (41.42)
KPS	<70	18 (5.54)	1 (0.31)	6 (4.51)	0 (0)	14 (1.66)	1 (0.12)	2 (0.65)	1 (0.32)
	≥70	260 (80.00)	46 (14.15)	106 (79.7)	21 (15.79)	457 (54.21)	371 (44.01)	177 (57.28)	129 (41.75)
CRP	≤10	58 (17.85)	8 (2.46)	34 (25.56)	12 (9.02)	367 (43.53)	325 (38.55)	135 (43.69)	118 (38.19)
	>10	220 (67.69)	39 (12.00)	78 (58.65)	9 (6.77)	104 (12.34)	47 (5.58)	44 (14.24)	12 (3.88)
Albumi	<40	262 (80.62)	39 (12.00)	102 (76.69)	16 (12.03)	330 (39.15)	168 (19.93)	120 (38.83)	54 (17.48)
	≥40	16 (4.92)	8 (2.46)	10 (7.52)	5 (3.76)	141 (16.73)	204 (24.20)	59 (19.09)	76 (24.60)
Total cholesterol	<3	29 (8.92)	1 (0.31)	11 (8.27)	1 (0.75)	21 (2.49)	3 (0.36)	8 (2.59)	3 (0.97)
	≥3	249 (76.62)	46 (14.15)	101 (75.94)	20 (15.04)	450 (53.38)	369 (43.77)	171 (55.34)	127 (41.10)

#### 3.2.2 Analysis of related factors with decreased serum iron in lung cancer patients

Univariate analysis shows that the differences in whether age, sex, different cancer staging, surgery, and albumin will cause the occurrence of CRA in lung cancer patients with decreased serum iron have statistical significance. Taking the results of univariate analysis with *p* < 0.01 to select affecting factors as the independent variable and whether CRA occurs or not as the dependent variable, multivariate logistic regression is used to analyze the related factors of the occurrence of CRA in lung cancer patients with decreased serum iron to establish a logistic regression prediction model. The overall prediction accuracy of the model is 86.2%. The results show that whether CRA occurs or not in lung cancer patients with decreased serum iron is mainly related to albumin, age, and cancer staging (*p* < 0.05). See Table 5.

**TABLE 5:** Analysis of related factors in patients with reduced serum iron Levels in lung cancer.

Items	Univariate analysis		Multivariate analysis	
	OR (95% CI)	*p*	OR (95% CI)	*p*
CRP	1.35 (0.762 ~ 2.39)	0.304		
Albumin	3.309 (1.605 ~ 6.823)	<0.01**	2.618 (1.172 ~ 5.847)	<0.05*
Total cholesterol	3.771 (0.89 ~ 15.986)	0.072		
Gender	2.305 (1.367 ~ 3.886)	<0.01**	1.556 (0.875 ~ 2.766)	0.132
Age	0.386 (0.224 ~ 0.665)	<0.01**	0.533 (0.29 ~ 0.98)	<0.05*
Cancer staging	2.01 (1.609 ~ 2.511)	<0.01**	1.726 (1.33 ~ 2.239)	<0.01**
Pathological type: Adenocarcinoma		<0.01**		
Pathological type: Squamous carcinoma	14.124 (3.397 ~ 58.717)	<0.01**		
Pathological type: Small cell carcinoma	426485358.5 (0)	0.998		
Pathological type: Others	426485358.5 (0)	0.999		
Surgery	0.261 (0.148 ~ 0.461)	<0.01**	0.65 (0.322 ~ 1.316)	0.231
Radiotherapy	1.697 (0.7 ~ 4.111)	0.242		
Chemotherapy	1.686 (0.915 ~ 3.107)	0.094		
Targeted therapy	1.041 (0.541 ~ 2.001)	0.905		
Immunotherapy	0.442 (0.167 ~ 1.173)	0.101		
KPS	4.393 (0.584 ~ 33.029)	0.15		

* *p* < 0.05.

** *p* < 0.01.

#### 3.2.3 Analysis of factors in patients with normal serum iron levels in lung cancer

Univariate analysis revealed statistically significant differences in the impact of age, gender, cancer staging, surgery, radiation therapy, CRP, albumin, and total cholesterol on the incidence of CRA in lung cancer patients with normal serum iron levels. Using a significance threshold of *p* < 0.01, factors identified through univariate analysis were selected as independent variables, with the incidence of CRA as the dependent variable. Multivariate logistic regression analysis was then employed to examine the factors influencing CRA incidence among these patients, culminating in the development of a logistic regression prediction model. This model exhibited an overall prediction accuracy of 68.2%. Analysis indicated that CRP, albumin, total cholesterol, and cancer staging were primarily associated with CRA incidence in lung cancer patients with normal serum iron levels (*p* < 0.05). Refer to Table 6 for detailed information.

**TABLE 6:** Analysis of related factors in patients with normal serum iron in lung cancer.

Items	Univariate analysis		Multivariate analysis	
	OR (95% CI)	*p*	OR (95% CI)	*p*
CRP	2.214 (1.595 ~ 3.072)	<0.01**	1.444 (1.004 ~ 2.077)	<0.05*
Albumin	2.838 (2.227 ~ 3.616)	<0.01**	2.235 (1.719 ~ 2.906)	<0.01**
Total cholesterol	3.86 (1.59 ~ 9.372)	<0.01**	3.29 (1.286 ~ 8.419)	<0.05*
Gender	1.551 (1.227 ~ 1.961)	<0.01**	1.042 (0.799 ~ 1.358)	0.762
Age	0.625 (0.485 ~ 0.807)	<0.01**	0.766 (0.58 ~ 1.011)	0.06
Cancer staging	1.682 (1.523 ~ 1.857)	<0.01**	1.52 (1.353 ~ 1.709)	<0.01**
Pathological type: Adenocarcinoma		<0.01**		
Pathological type: Squamous carcinoma	0.374 (0.12 ~ 1.169)	0.091		
Pathological type: Small cell carcinoma	0.708 (0.217 ~ 2.311)	0.568		
Pathological type: Others	3.778 (0.736 ~ 19.382)	0.111		
Surgery	0.363 (0.275 ~ 0.48)	<0.01**	0.759 (0.546 ~ 1.056)	0.102
Radiotherapy	1.738 (1.175 ~ 2.569)	<0.01**	1.02 (0.661 ~ 1.573)	0.929
Chemotherapy	0.749 (0.58 ~ 0.968)	0.027		
Targeted therapy	1.327 (0.96 ~ 1.832)	0.086		
Immunotherapy	2.132 (1.022 ~ 4.447)	0.044		
KPS	6.309 (1.444 ~ 27.568)	0.014		

* *p* < 0.05.

** *p* < 0.01.

#### 3.2.4 Development of a nomogram prediction model

**FIGURE 2:**
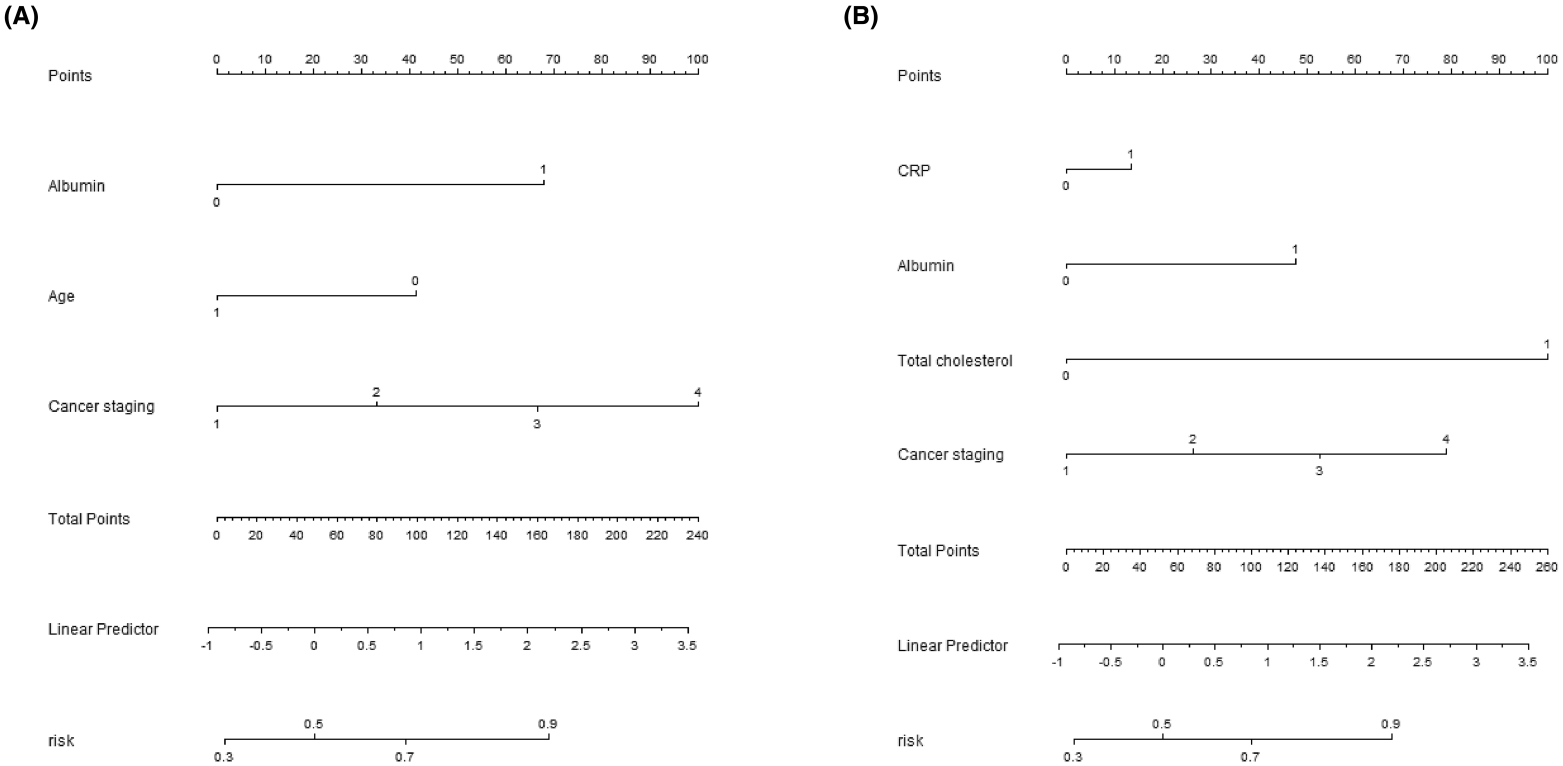
Nomogram prediction model of CRA in patients with decreased serum iron and patients with normal serum iron in lung cancer. (A) Nomogram prediction model of CRA in lung cancer patients with decreased serum iron.(B) nomogram prediction model of CRA in lung cancer patients with normal serum iron.

The influencing factors identified through the analysis of lung cancer patients with either decreased or normal serum iron levels were utilized to create nomogram prediction models for CRA incidence in these two patient groups. In each nomogram, variables were assigned scores on a top score line. The total score, derived by summing the scores of all variables, corresponds to the predicted probability of anemia. See Figure 2 for the nomograms.

#### 3.2.5 Model testing and evaluation

**FIGURE 3:**
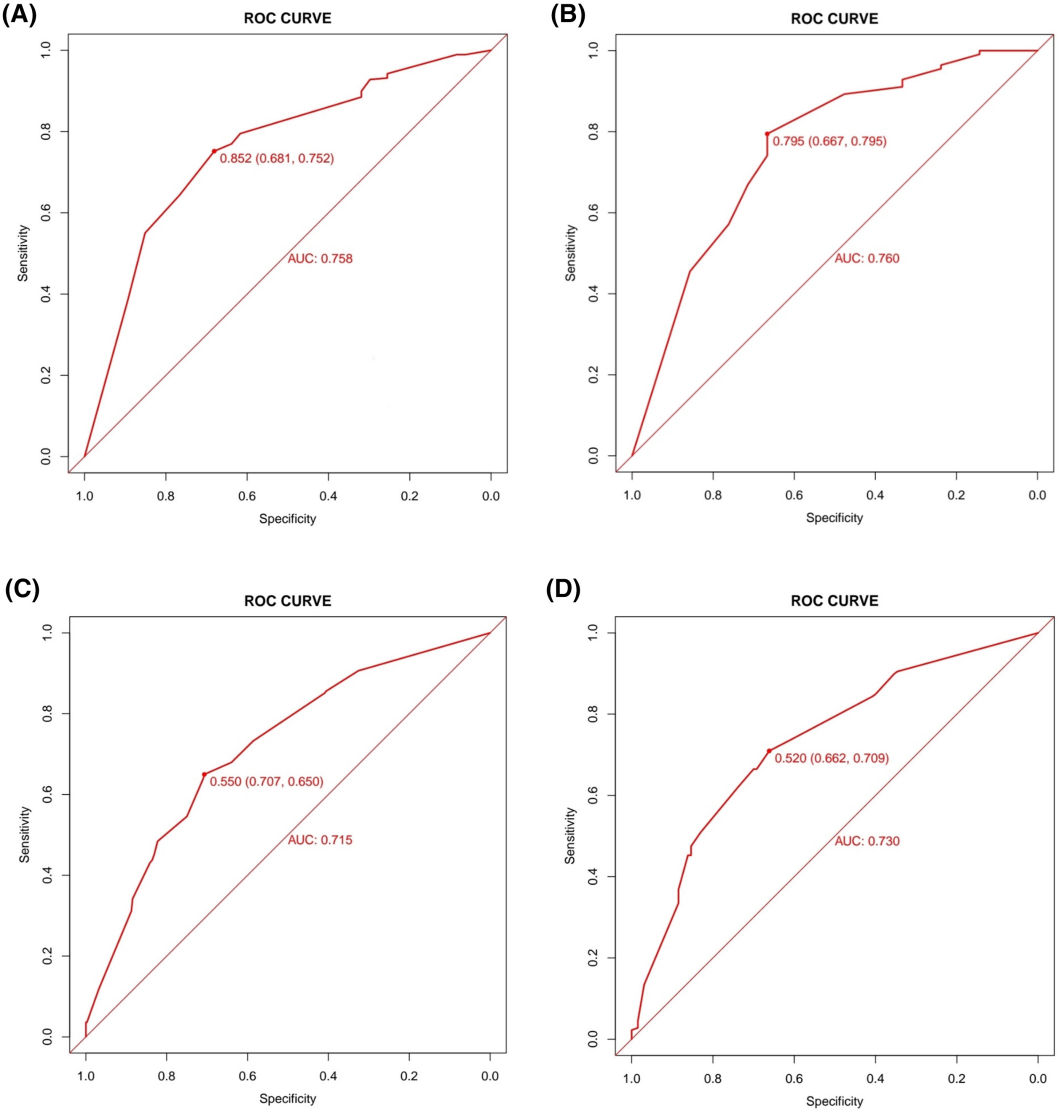
ROC curve of the nomogram prediction model about the occurrence of CRA in lung cancer patients with decreased serum iron and normal serum iron. (A) Training Cohort ROC curve of the nomogram prediction model about the occurrence of CRA in lung cancer patients with decreased serum iron. (B) Validation cohort ROC curve of the nomogram prediction model about the occurrence of CRA in lung cancer patients with decreased serum iron. (C) Training cohort ROC curve of the nomogram prediction model about the occurrence of CRA in lung cancer patients with normal serum iron. (D) Validation cohort ROC curve of the nomogram prediction model about the occurrence of CRA in lung cancer patients with normal serum iron.

**FIGURE 4:**
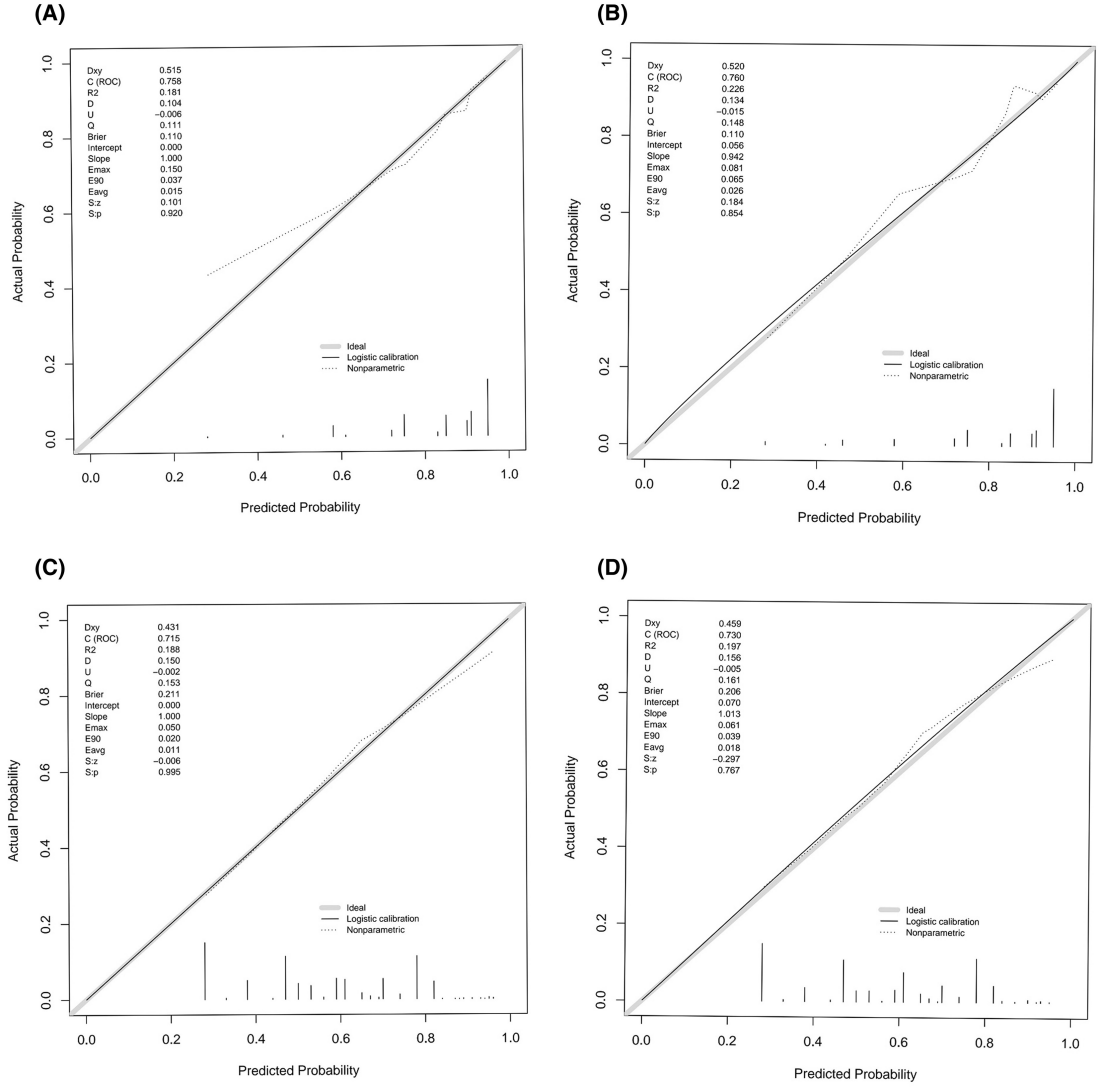
Calibration curve of the nomogram prediction model about the occurrence of lung cancer patients with decreased serum iron and normal serum iron. (A) Training cohort calibration curve of the nomogram prediction model about the occurrence of CRA in lung cancer patients with decreased serum iron. (B) Validation cohort calibration curve of the nomogram prediction model about the occurrence of CRA in lung cancer patients with decreased serum iron. (C) Training cohort calibration curve of the nomogram prediction model about the occurrence of CRA in lung cancer patients with normal serum iron. (D) Validation cohort calibration curve of the nomogram prediction model about the occurrence of CRA in lung cancer patients with normal serum iron.

For the nomogram predicting CRA incidence in lung cancer patients with decreased serum iron, the area under the ROC curve was determined from multivariate logistic regression analysis results. The area under the ROC curve for both the training and validation cohorts was 0.758 and 0.760, respectively (refer to Figure 3A,B). Similarly, for the nomogram predicting CRA incidence in lung cancer patients with normal serum iron, areas under the ROC curve were calculated, resulting in values of 0.715 and 0.730 for the training and validation cohorts, respectively (refer to Figure 3C,D). Comparison of ROC curves for both models demonstrated their effective discriminative power. Calibration curves indicated that both prediction models were well‐calibrated (refer to Figure 4).

#### 3.2.6 Clinical utility analysis of the prediction model

**FIGURE 5:**
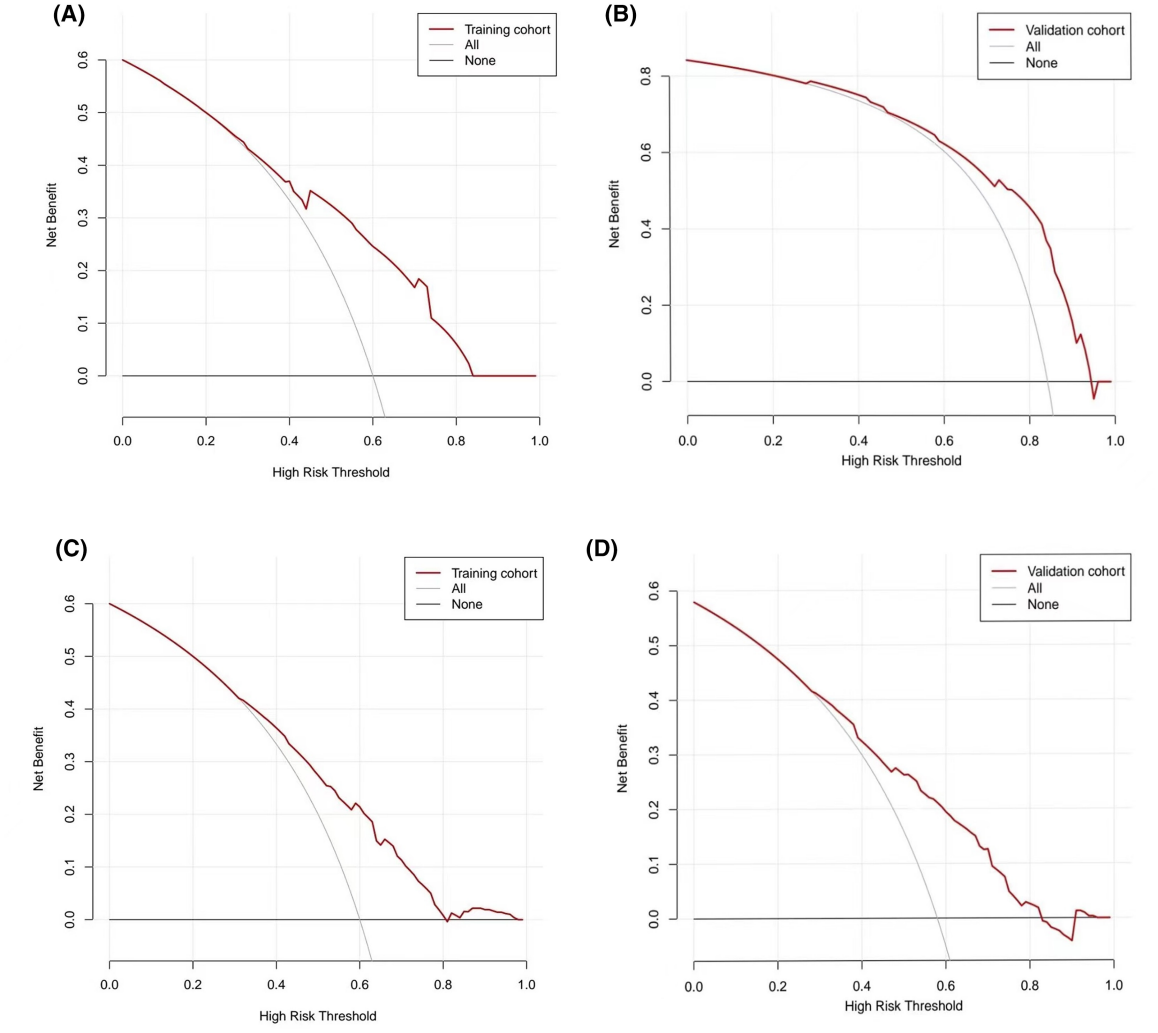
DCA curve of the nomogram prediction model about the occurrence of CRA in lung cancer patients with decreased serum iron and normal serum iron. (A) Training cohort DCA curve of the nomogram prediction model about the occurrence of CRA in lung cancer patients with decreased serum iron. (B) Validation cohort DCA curve of the nomogram prediction model about the occurrence of CRA in lung cancer patients with decreased serum iron. (C) Training cohort DCA curve of the nomogram prediction model about the occurrence of CRA in lung cancer patients with normal serum iron. (D) Validation cohort DCA curve of the nomogram prediction model about the occurrence of CRA in lung cancer patients with normal serum iron.

The clinical usefulness of the nomogram prediction models was assessed using DCA. DCA results showed that the risk thresholds for the training cohorts of the nomogram models predicting CRA incidence in lung cancer patients with decreased and normal serum iron were 0.3–0.85 and 0.3–0.8, respectively. For the validation cohorts, the risk thresholds were 0.3–0.95 and 0.3–0.83, respectively. Additionally, the net benefit of clinical intervention based on the nomogram models surpassed that of the no‐intervention approach, indicating the models' significant clinical efficacy (refer to Figure 5).

## 4 DISCUSSION

CRA is a prevalent complication of malignant tumors that significantly impacts a patient's prognosis. At present, there are few studies on the influencing factors of CRA in lung cancer. Therefore, it is crucial to actively investigate the related influencing factors of CRA to prevent and treat the disease efficiently. In this study, the influencing factors were screened by univariate and multivariate logistic regression analyses, and nomogram models were constructed to evaluate the risk of CRA in patients with different serum iron levels in lung cancer. We found that lung cancer CRA is primarily related to the patient's past surgeries, chemotherapy, KPS score, serum iron, CRP, albumin, and total cholesterol levels through the univariate analysis of clinical data and laboratory indices of 1040 lung cancer patients with CRA. Surgery can lead to anemia due to the trauma associated with lung cancer surgery, intraoperative bleeding, and other factors that increase the likelihood of anemia.^14^ Chemotherapy is another common cause of anemia, as it can lead to bone marrow suppression and cause gastrointestinal reactions such as nausea and vomiting, resulting in anemia.^15^ The KPS score is used to assess a patient's general health condition and forms the basis for their antitumor treatment plan. Malignant tumors are high‐consumption and high‐metabolic diseases that lead to malnutrition, resulting in low serum albumin, total cholesterol levels, and a decrease in raw materials for erythropoiesis such as iron, vitamin B12, and folic acid, thus leading to anemia. Additionally, as the malignancy progresses, patients experience various accompanying symptoms, leading to an inflammatory state in the body and an increase in inflammatory factors. Tumor‐related inflammation may interfere with nutrition and hamper hematopoiesis, leading to anemia.^16^, ^17^

It is worth noting that iron deficiency is a common cause of anemia in patients with malignant tumors. To evaluate iron deficiency, ferritin and transferrin saturation are often used in clinical settings, with ferritin being the most sensitive laboratory index. In general, the lower the ferritin level, the higher the chances of having iron‐deficiency anemia. However, in specific situations, such as inflammation or malnutrition, ferritin may falsely increase,^18^, ^19^ seriously impacting the diagnosis of iron deficiency and consequently affecting anemia. Therefore, it is crucial to explore the impact of serum iron on anemia actively. Serum iron is an essential component of Hb synthesis in vivo and often maintains its dynamic balance through erythrocyte programming and hepcidin regulation.^20^, ^21^, ^22^ Although it cannot evaluate the body's iron storage or metabolism ability, it can reflect the iron concentration in vivo. When iron metabolism and homeostasis are disrupted in patients with malignant tumors, red blood cells may suffer from insufficient iron supply, leading to anemia.^23^ Iron deficiency leads to reduced Hb synthesis in red blood cells, smaller red blood cell volume, and decreased oxygen‐carrying capacity, ultimately resulting in iron‐deficiency anemia.^24^ Although serum iron cannot serve as the diagnostic standard for iron deficiency, it can be a good predictor of iron‐deficiency anemia. Therefore, determining the effect of different serum iron levels on CRA in lung cancer patients and diagnosing and treating iron deficiency in early stages may be critical in managing CRA effectively.

This study utilizes multivariate logistic regression analysis to identify the related influencing factors that affect the occurrence of CRA in lung cancer patients with decreased serum iron, which include albumin, age, and cancer staging. Additionally, the related influencing factors for patients with normal serum iron are found to be CRP, albumin, total cholesterol, and cancer staging. Based on these factors, nomogram prediction models for CRA are constructed for both patient groups, demonstrating good prediction efficiency and clinical practicality. Previous studies have shown that serum iron levels are negatively correlated with tumor staging and positively correlated with patient prognosis in lung cancer cases,^25^ as the incidence of CRA in lung cancer patients tends to increase with later stages of cancer and lower levels of serum iron due to tumor cell proliferation and increased demand for iron. Malignant tumors are consumptive diseases, and with the progression of the disease, some patients with advanced lung cancer consume more in the body because of the deterioration of malignant fluid, which leads to the decrease in serum iron content of hematopoietic raw materials, thus leading to CRA. Moreover, different pathological types are also closely related to disease progression. For example, adenocarcinoma is prone to distant metastasis, and squamous carcinoma is prone to hemoptysis, and disease progression will also aggravate anemia.^26^ Therefore, the degree of disease progression can also be used as an independent risk factor for CRA.^27^ Furthermore, age is also identified as an important risk factor for CRA among lung cancer patients, with elderly patients being more susceptible due to decreased hematopoietic raw material absorption, erythropoietin (EPO) secretion, and bone marrow erythropoiesis.^28^, ^29^

It is of great significance to evaluate the effects of serum albumin, total cholesterol, and CRP on serum iron. Serum albumin and total cholesterol are effective indicators to evaluate the nutritional status of the body, and the decrease in their levels reflects malnutrition. CRP can be used as one of the most valuable indicators to evaluate the inflammatory state of cancer patients,^30^ and its elevated level reflects the inflammatory state of the body. Iron deficiency in lung cancer is mainly manifested in the decrease of serum iron level, which is related to the decrease in iron storage and iron metabolism disorder. The decrease in iron storage is closely related to malnutrition, digestive system surgery, and chronic blood loss, and iron metabolism disorder is mainly related to chronic inflammation.^31^

The findings of this study further demonstrate that CRP is a significant influencing factor for CRA in patients with normal serum iron in lung cancer, which means that the inflammatory state represented by CRP can affect CRA. Studies have confirmed that when the body is in an inflammatory state, the release of inflammatory factors can be regarded as an important cause of CRA,^32^ which mainly shows that proinflammatory cytokines can promote the secretion of inflammatory factors, such as interleukin‐1 (IL‐1), interleukin‐6 (IL‐6), interferon, and tumor necrosis factor (TNF), etc., which can not only inhibit the production of red blood cells but also promote their apoptosis, thus leading to inflammatory anemia or chronic disease.^33^, ^34^ At the same time, the release of inflammatory cytokines is related to the production of reactive oxygen species, which can inhibit the production of red blood cells, interfere with nutritional status, and reduce the raw materials for red blood cell production, such as iron, vitamin B12, and folic acid, thus aggravating anemia.^17^ Moreover, reactive oxygen species can inhibit the immune response mediated by CD8+ T cells, which can contribute to the occurrence of anemia.^35^

CRP is a risk factor that can cause the occurrence of CRA in lung cancer patients with normal serum iron; however, CRP has no significant effect on CRA in lung cancer patients with decreased serum iron, which may suggest that serum iron has a unique position in the process of CRA caused by inflammation. Previous studies have shown that CRP levels in patients with lung cancer are negatively correlated with serum iron levels. CRP can stimulate the synthesis of ferritin and promote its combination with intracellular iron, thus reducing the serum iron content in the body and increasing the risk of CRA and iron deficiency.^36^ In addition, the increase in CRP means that the body is in an inflammatory state, and inflammatory cytokines can stimulate the liver to synthesize hepcidin,^37^ which can not only bind with iron transport‐related proteins on the surface of duodenal cells to weaken its effect of transferring iron but also regulate the transport process of iron through intestinal mucosa, inhibit the absorption of iron and the release of iron in cells, resulting in iron metabolism disorder and eventually leading to anemia.^38^, ^39^, ^40^ The above results show that CRP and the inflammatory state represented by CRP can lead to a decrease in serum iron and further contribute to anemia.

However, in patients with lung cancer whose serum iron level has already decreased, the iron metabolism and iron homeostasis in the body have been disordered. Considering the dynamic balance and negative feedback mechanism of the body, whether CRP and the inflammatory state represented by CRP will further aggravate the decrease in serum iron levels, thus affecting anemia, has no research to support at present and is worth further discussion.

Since this study is a single‐center and retrospective study with a relatively small sample size, the established model has not been verified in other institutions, and there may be some bias. Therefore, it is necessary to carry out larger sample size and multicenter, prospective studies in the future to further improve the results of this study and guide clinical treatment.

## 5 CONCLUSION

In summary, chemotherapy, KPS score, serum iron, CRP, albumin, and total cholesterol are all factors related to CRA in lung cancer patients. Specifically, albumin, age, and cancer staging are related factors that impact the occurrence of CRA in lung cancer patients with decreased serum iron. On the other hand, CRP, albumin, total cholesterol, and cancer staging are factors that impact the occurrence of CRA in lung cancer patients with normal serum iron. We also discovered that CRP is a risk factor for the development of CRA in lung cancer patients with normal serum iron, but it is not a contributing factor for the reduction of CRA in patients with decreased serum iron levels. Therefore, whether CRP and the inflammatory state represented by CRP will further aggravate the decrease in serum iron levels, thus contributing to anemia, warrants further study.

## AUTHOR CONTRIBUTIONS

**Quanyao Li:** Data curation (lead); methodology (lead); resources (equal); writing – original draft (lead). **Wen‐xiao Yang:** Data curation (equal); funding acquisition (lead); methodology (equal); resources (equal); writing – original draft (equal). **Hui Liu:** Formal analysis (equal); methodology (equal); software (lead); visualization (lead). **Jialin Yao:** Formal analysis (equal); methodology (equal); software (equal); visualization (equal). **Qin Wang:** Conceptualization (equal); formal analysis (equal); investigation (equal). **Dan Lin:** Conceptualization (equal); formal analysis (equal); investigation (equal). **Jun Shi:** Project administration (equal); supervision (lead); validation (lead); writing – review and editing (lead).

## FUNDING INFORMATION

This work was supported by National Natural Science Foundation of China (No. 82205213).

## CONFLICT OF INTEREST STATEMENT

The authors have no relevant financial or nonfinancial interests to disclose.

## ETHICS STATEMENT

Study that has been written consent by the Ethics Review Committee of Yueyang Integrated Traditional Chinese and Western Medicine Hospital affiliated to Shanghai University of Traditional Chinese Medicine (Project number: 2023–077). The requirement for written informed consent was waived by the Ethics Review Committee of Yueyang Integrated Traditional Chinese and Western Medicine Hospital affiliated to Shanghai University of Traditional Chinese Medicine because of the retrospective nature of the study.

## Data Availability

All data generated or analyzed during this study are included in this published article.
